# The combination of calreticulin-targeting L-ASNase and anti-PD-L1 antibody modulates the tumor immune microenvironment to synergistically enhance the antitumor efficacy of radiotherapy

**DOI:** 10.7150/thno.90376

**Published:** 2024-01-20

**Authors:** Ying Zhang, Venu Akhil, Ho Seong Seo, Hae Ran Park, Soo Hyun Kim, Sung-Hwan You, Zhipeng Liu, So-young Kim, Rukhsora D. Sultonova, Jung-Joon Min, Yeongjin Hong

**Affiliations:** 1 Institute for Molecular Imaging and Theranostics, Department of Nuclear Medicine, Chonnam National University Medical School and Hwasun Hospital, Jeollanam-do, Republic of Korea.; 2 Research Division for Radiation Science, Korea Atomic Energy Research Institute, Jeollabuk-do, Republic of Korea.; 3 Department of Microbiology, Chonnam National University Medical School, Jeollanam-do, Republic of Korea.; 4 Department of Laboratory Medicine, Chonnam National University Medical School and Chonnam National University Hospital, Gwangju, Republic of Korea.; 5 CNCure Biotech, Inc., Jeollanam-do, Republic of Korea.; 6 Brain Tumor Research Laboratory, Biomedical Research Institute, Chonnam National University Hwasun Hospital, Jeollanam-do, Republic of Korea.; 7 New Uzbekistan University, Tashkent, Uzbekistan.; 8 Republican Oncology Research Center Tashkent Region Branch, Tashkent, Uzbekistan.; 9 Current affiliation: State Key Laboratory of Drug Research, Molecular Imaging Center, Shanghai Institute of Materia Medica, Chinese Academy of Sciences, Shanghai, 201203, China.

**Keywords:** Cancer radiotherapy, immunogenic cell death, calreticulin, L-ASNase, reactive oxygen species, immune checkpoint inhibitor

## Abstract

Radiotherapy (RT) triggers immunogenic cell death (ICD). L-ASNase, which catalyzes the conversion of asparagine (Asn), thereby depleting it, is used in the treatment of blood cancers. In previous work, we showed that CRT3LP and CRT4LP, PASylated L-ASNases conjugated to the calreticulin (CRT)-specific monobodies CRT3 and CRT4, increase the efficacy of ICD-inducing chemotherapy. Here, we assessed their efficacy in tumor-bearing mice treated with RT.

**Methods:** Monobody binding was evaluated by *in silico* molecular docking analysis. The expression and cellular localization of ecto-CRT were assessed by confocal imaging and flow cytometry. The antitumor effect and the roles of CRT3LP and CRT4LP in irradiation (IR)-induced ICD in tumors were analyzed by ELISA, immunohistochemistry, and immune analysis methods.

**Results:** Molecular docking analysis showed that CRT3 and CRT4 monobodies were stably bound to CRT. Exposure to 10 Gy IR decreased the viability of CT-26 and MC-38 tumor cells in a time-dependent manner until 72 h, and increased the expression of the ICD marker ecto-CRT (CRT exposed on the cell surface) and the immune checkpoint marker PD-L1 until 48 h. IR enhanced the cytotoxicity of CRT3LP and CRT4LP in CT-26 and MC-38 tumor cells, and increased reactive oxygen species (ROS) levels. In mice bearing CT-26 and MC-38 subcutaneous tumors treated with 6 Gy IR, Rluc8-conjugated CRT-specific monobodies (CRT3-Rluc8 and CRT4-Rluc8) specifically targeted tumor tissues, and CRT3LP and CRT4LP increased total ROS levels in tumor tissues, thereby enhancing the antitumor efficacy of RT. Tumor tissues from these mice showed increased mature dendritic, CD4^+^ T, and CD8^+^ T cells and pro-inflammatory cytokines (IFNγ and TNFα) and decreased regulatory T cells, and the expression of tumor cell proliferation markers (Ki67 and CD31) was downregulated. These data indicate that the combination of IR and CRT-targeting L-ASNases activated and reprogramed the immune system of the tumor microenvironment. Consistent with these data, an immune checkpoint inhibitor (anti-PD-L1 antibody) markedly increased the therapeutic efficacy of combined IR and CRT-targeting L-ASNases.

**Conclusion:** CRT-specific L-ASNases are useful as additive drug candidates in tumors treated with RT, and combination treatment with anti-PD-L1 antibody increases their therapeutic efficacy.

## Introduction

Radiotherapy (RT) using high-energy beam irradiation (IR) is an effective cancer treatment 1. The basic principles of RT are well known 2. IR causes either single strand or double strand breaks in genomic DNA, thereby arresting cell division. In addition, IR generates cytotoxic reactive oxygen species (ROS) such as superoxide anions (O_2_^.-^), hydroxyl radicals (HO**^.^**), and hydrogen peroxide (H_2_O_2_).

ROS are produced during normal cellular metabolic processes and act as “redox messengers” 3. Increased ROS levels activate signaling molecules such as JNK, p38 MAPK, and NADPH oxidase to induce apoptosis 4. Excess ROS upregulate manganese-dependent superoxide dismutase (MnSOD/SOD_2_), an antioxidant enzyme that converts O_2_^.-^ to H_2_O_2_ in the mitochondria. Subsequently, H_2_O_2_ is converted to H_2_O by detoxifying enzymes such as catalases, peroxidases, or peroxiredoxins 5. Therefore, upregulation of SOD_2_ protects cells against oxidative stress and increases radio-resistance 6. O_2_^.-^ downregulates SOD_2_ via IR-delayed mitochondrial O_2_^.-^ clearance and increases oxidative stress level 7, 8.

RT has been known to trigger immune responses; it increases ROS levels, thereby inducing the maturation of dendritic cells (DCs) and activating cytotoxic T lymphocytes (CTLs) 9-11. RT also promotes immunogenic cell death (ICD) 12. IR-treated tumor cells show increased expression of damage-associated molecular patterns (DAMPs), such as translocation of CRT on the cell surface (ecto-CRT) and extracellular release of HGMB1 and ATP 13. RT activates immune responses against cancer cells, thereby exerting “abscopal effects” against untreated tumors 14-16.

L-asparaginase (L-ASNase) is a bacterial enzyme that converts L-asparagine (Asn) to L-aspartic acid (Asp) and ammonia 17. Despite the side effects of L-ASNase, such as increased immunogenicity and hypersensitivity, it has been used clinically for many years to treat blood cancers such as lymphoblastic leukemia 18, 19. Tumor cells have high nutrient requirements to support their increased proliferation rate, and L-ASNase depletes Asn, decreasing its bioavailability to inhibit tumor cell growth and proliferation. In addition, L-ASNase causes metabolic stress, which induces ROS accumulation in cancer cells, leading to mitochondrial dysfunction and Ca^2+^ release, ultimately triggering programmed cell death 20-22. This leads to tumor cell death via autophagy and p53/SOD_2_/ROS-mediated apoptotic pathways 23, 24. However, L-ASNase lacks targeting activity, and it is thus ineffective against solid tumors 25, 26. Strategies to induce L-ASNase to target solid tumors have been reported. An urchin-like helical polypeptide-ASNase conjugate effectively inhibited the growth of human NK/T lymphoma NKYS solid tumors 27. Attenuated *Salmonella typhimurium* was engineered to secrete L-ASNase 28. The bacterium targets various solid tumors and efficiently suppresses tumor growth. Tumor cell sensitivity to L-ASNase is correlated with the expression levels of Asn synthetase (ASNS) 29-31, although these findings are controversial. Supraphysiological or physiological concentrations of Asn prevent its *de novo* biosynthesis in high-ASNS B cell lymphoma regardless of ASNS expression levels 32, and L-ASNase possesses dual activity as an asparaginase and glutaminase 33.

Immune checkpoint inhibitors (ICIs) are antibody-based drugs that block antitumor immune responses 34. Of these, PD-1 and PD-L1 have been studied extensively. PD-L1 is expressed in many cancers as well as by macrophages 35, 36. The interaction between PD-1 and PD-L1 on activated T cells triggers apoptosis, anergy, exhaustion, and an unchecked immune response. Therefore, inhibiting binding between PD-1 and PD-L1 increases antitumor immune responses 37. The USA FDA have approved the use of antibodies against PD-1 and PD-L1 in the treatment of solid tumors such as lung cancer 38. However, only a fraction of cancer patients responds to ICI-mediated cancer immunotherapy, and additional strategies need to be developed to optimize treatment regimens 39, 40. For instance, combinations of ICI-mediated cancer immunotherapy with chemotherapy 41, 42, RT 43, 44, and bacteria-mediated tumor therapy have been explored 45-47.

**Scheme 1:**
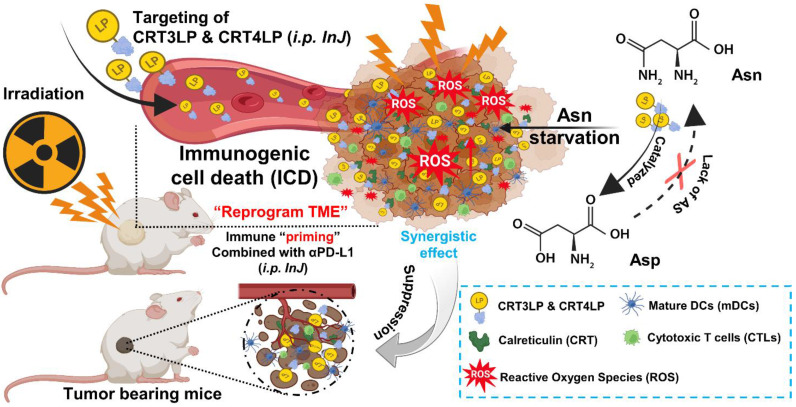
** Schematic illustration of the antitumor effects of the combination of CRT-targeting 76 L-ASNases and αPD-L1 in IR-treated mice.** TME, tumor microenvironment.

In a previous study, we developed CRT-targeting monobodies (CRT3 and CRT4) based on the human fibronectin domain III (FN3) scaffold 48. These monobodies specifically target ecto-CRT in solid tumors of mice treated with ICD-inducing chemotherapy. Additionally, we generated PASylated L-ASNases conjugated with CRT-specific monobodies (CRT3LP and CRT4LP), which increased the therapeutic efficacy of chemotherapy 49. Here, we investigated whether CRT-targeting L-ASNases can increase the therapeutic efficacy of RT by activating immune responses. We also examined the potential synergistic effect of anti-PD-L1 antibody (αPD-L1) in combination with RT and CRT-targeting L-ASNases therapy. A potential mechanism underlying the antitumor activity of IR, CRT-targeting L-ASNases, and αPD-L1 is shown in **Scheme 1**.

## Methods

### Cell lines and reagents

Murine colon carcinoma CT-26 and MC-38 cell lines were purchased from the American Type Culture Collection (ATCC), USA, and Kerafast, USA, respectively. DMEM medium, fetal bovine serum (FBS), Pierce™ high-capacity endotoxin removal spin columns, and phosphate-buffered saline (PBS) were purchased from Gibco/Thermo Fisher Scientific, USA. Penicillin/streptomycin solution and crystal violet reagent were purchased from Sigma-Aldrich, USA. The cells were cultured as previously described 47. The ASNase activity assay kit, rabbit-specific HRP/DAB (ABC) detection IHC kit, and recombinant CRT were purchased from Abcam, USA. L-ASNase was purchased from Prospec, USA. Cell Counting Kit-8 (CCK-8) and ROS-ID® Total ROS detection kits were purchased from Enzo Life Sciences, USA. Coelenterazine was purchased from Biotium, USA. MojoSort™ mouse pan DC isolation kit was purchased from BioLegend, USA. Mouse TNF-α and IFN-γ ELISA kits were from Invitrogen/Thermo Fisher Scientific. All antibodies used in this study, and their sources, are listed in **Table 1**.

### Protein purification

*E. coli* BL21(DE3) strains transformed with expression plasmids (pETh-CRT3-LASP-PAS200, pETh-CRT4-LASP-PAS200, or pETh-Fn3(DGR)-LASP-PAS200) were used to purify PASylated CRT-targeting L-ASNases (CRT3LP, CRT4LP, or #DGRLP) as described 49. *E. coli* BL21(DE3) strains transformed with expression plasmids pETh-CRT3-Rluc8, pETh-CRT4-Rluc8, or pETh-Fn3(DGR)-Rluc8 were used to purify *Renilla* luciferase variant 8 (Rluc8)-conjugated monobodies (CRT3-Rluc8, CRT4-Rluc8, or #DGR-Rluc8) as described 48. The conjugated proteins were expressed in the above bacterial strains and purified using His GraviTrap columns (GE Healthcare, USA) and PD-10 desalting column (GE Healthcare, USA), as previously described 49. Endotoxin contamination was removed from the purified proteins using high-capacity endotoxin removal spin columns (Thermo Fisher Scientific, USA). Finally, protein concentration and ASNase activity were measured with an asparaginase activity assay kit and an OxiRed probe (Abcam, USA) 49. The specific activity of CRT-targeting L-ASNases was similar to that published previously (i.e., ∼6.5 IU/nmol of each monobody) 49.

### *In silico* molecular docking analysis

The amino acid sequences of the monobodies (CRT3 and CRT4) and the control (#DGR) were described previously 48. Information about the CRT sequence (PDB:1HHN) was obtained from the PDB database (https://www.rcsb.org/). To assess binding between CRT and monobodies, the CRT and monobody sequences were submitted to HDOCK (http://hdock.phys.hust.edu.cn/) and all parameters were chosen with default options. The models with the highest-quality docking score were chosen for analysis and visualized with PyMol 2.2.0. software (Schrödinger, NY, USA). The functional residues involved in hydrogen bonding interactions, salt bridges, and hydrophobic interactions were evaluated by LigPlot^+^ 2.2.4 software (EMBL-EBI, Cambridge, UK). CRT and monobodies are depicted as blue-violet and cyan, respectively, in visualized models. The binding sites are depicted in pink. Additionally, the binding energy between CRT and the monobodies was calculated by Prodigy software (https://bianca.science.uu.nl/prodigy/).

### Flow cytometry analysis of IR-treated tumor cells

To measure the expression of ecto-CRT on RT-treated CT-26 and MC-38 cells (1 × 10^5^), cells were cultured in dishes for 1 day, treated with 10 Gy (3.3 Gy/min) IR delivered by a Gammacell 3000 Elan irradiator (Best Theratronics Ltd., Ottawa, Canada), and further cultured for 0-72 h. Next, cells were detached from dishes by scraping, washed three times with PBS, and fixed for 15 min with 1% paraformaldehyde (PFA; Biosesang, South Korea). The cells were then stained with a PE-conjugated anti-CRT antibody (1:50 dilution) for 1 h on ice.

To measure changes in PD-L1 expression in RT-treated cells, tumor cells were cultured for 48 h after treatment and stained with a PE-conjugated αPD-L1 antibody (5 μg/mL).

To measure specific binding of CRT-targeting L-ASNases to ecto-CRT, CT-26 and MC-38 cells were treated with 10 Gy (3.3 Gy/min) IR, detached from the dishes after 48 h, and fixed for 15 min with 1% PFA prior to staining with 100 nM of CRT-targeting L-ASNases (CRT3LP, CRT4LP, or #DGRLP) for 1 h on ice. For the blocking assay, the cells were pre-incubated with anti-CRT monoclonal antibody (1:1000 dilution) for 1 h on ice, followed by incubation with CRT-targeting L-ASNases. The cells were then washed thoroughly with PBS and stained for 1 h with rabbit anti-His tag monoclonal antibody (1:1000 dilution), followed by Alexa Fluor 488-conjugated secondary antibody (5 μg/mL) for 1 h. The fluorescence signal was detected using a FACSCanto II Flow Cytometer (BD Biosciences, USA).

### Confocal immunofluorescence imaging

To visualize binding of CRT-targeting L-ASNases to IR-treated tumor cells, CT-26 and MC-38 cells (1 × 10^4^) were cultured on chamber slides (Thermo Scientific, USA) for 48 h and then treated with 10 Gy IR. At 48 h post-IR, the cells were fixed for 15 min with 1% PFA and washed with PBS containing 1% FBS. Then, the cells were stained with 100 nM CRT-targeting L-ASNases for 1 h on ice. After washing thoroughly with PBS containing 1% FBS, the cells were incubated for 1 h with anti-His tag monoclonal antibody (1:1000 dilution), followed by Alexa Fluor 488-conjugated secondary antibody (5 μg/mL) for 1 h. Cell membranes were stained with wheat AF555-conjugated wheat germ agglutinin (WGA) (1:5000 dilution). Finally, the cells were mounted in DAPI solution (Thermo Fisher Scientific, USA) to stain the nuclei. Immunofluorescence signals were detected using a LSM510 confocal microscope (ZEISS, Germany) and analyzed by ZEN-LSM imaging software (ZEISS, Germany).

### Cell viability

CT-26 and MC-38 cells (1 × 10^4^) were seeded into 96-well plates (n = 3) and cultured at 37 °C. The next day, cells were treated with or without 10 Gy IR and cultured for 24 h. The culture medium was replaced with fresh medium, and CRT-targeting L-ASNases (1 IU/mL) were added to the wells at 37 °C. After 24 h, cells were washed three times with fresh medium. Cell viability was measured using a CCK-8 kit.

### Analyses of ROS generation and SOD_2_ expression

CT-26 and MC-38 cells (5 × 10^5^/well) were seeded into 12-well plates and cultured overnight. The cells were then treated with or without 10 Gy IR and cultured for 24 h, followed by replacement of the medium with fresh medium. Next, CRT-targeting L-ASNases (1 IU/mL) were added to the wells. After 24 h, the cells were washed three times with fresh medium. Total intracellular ROS levels were measured with a ROS-ID® Total ROS detection kit. The cells were detached by scraping, washed three times with PBS, resuspended in 500 μL of ROS detection solution, and incubated in the dark at 37 °C for 30 min. Fluorescence signal intensity was measured at 488 nm using a flow cytometer.

SOD_2_ protein levels were evaluated by western blotting using cells treated as described above. Briefly, cells were dissolved in protein extraction solution (iNtRON Biotechnology, Korea), and the concentration of total protein was measured using BCA protein assay kits (Thermo Fisher Scientific). Proteins (10 μg/well) were separated in 12% SDS-PAGE gels and blotted on nitrocellulose membranes (Bio-Rad, Hercules, CA, USA), which were then stained with rabbit anti-SOD_2_/MnSOD antibody (1:1000 dilution), followed by horseradish peroxidase (HRP)-conjugated anti-rabbit secondary antibody (1:2000 dilution). After stripping, the same membranes were stained with anti-β-actin antibody (1:1000 dilution). Specific bands were detected using a LAS-3000 chemiluminescence detection system (Fuji, Japan), and band intensity was analyzed by ImageJ software [National Institutes of Health (NIH), USA]. SOD_2_ levels were normalized to those of β-actin.

### Generation and treatment of tumor-bearing mice

All animal experiments were performed in accordance with the general principles and procedures outlined in the NIH guidelines 50, and all protocols were approved by the Animal Care and Use Committee of Chonnam National University (permit number: HCRL 16-001). Female mice (aged 6 weeks) were obtained from Orient Company, Korea. CT-26 and MC-38 tumor cells (1 × 10^6^ in 100 µL PBS) were transplanted subcutaneously (*s.c.*) into the right flank of BALB/c and C57BL/6 mice, respectively. When tumor volumes reached approximately 100 mm^3^ (Day 0), mice received a single dose of tumor-specific IR (6 Gy, 0.9 Gy/min) delivered by a Gammacell 40 Exactor (Nordion International Inc., Ottawa, Canada). CRT-targeting L-ASNases (8 IU per 100 μL PBS) were then injected intraperitoneally (*i.p.*) daily from Day 3 to Day 7. PBS was used as a control. CRT3-Rluc8 and CRT4-Rluc8 (60 μg/100 μL PBS) were injected intravenously (*i.v.*) via the tail vein on Day 5. #DGR-Rluc8 was used as a control for the monobodies. An αPD-L1 antibody (10 mg/kg body weight) was injected intraperitoneally (*i.p.*) with 3-day interval, starting from Day 1. IgG2b isotype immunoglobulin was used as control for the αPD-L1 antibody. The length (L), width (W), and height (H) of each tumor were recorded every 3 days using a digital caliper, and tumor volume (mm^3^) was calculated using the formula (L × H × W)/2. Mice with tumors measuring approximately 1,500 mm^3^ were euthanized.

### *In vivo* and *ex vivo* bioluminescence imaging of Rluc8-conjugated monobodies

To obtain* in vivo* bioluminescence images, coelenterazine (400 ng/100 μL PBS) was injected *i.v.* into tumor-bearing mice treated with IR and Rluc8-conjugated CRT-specific monobodies on Day 5 plus 12 h, and images were acquired using the IVIS Lumina S5 imaging system (Perkin-Elmer, USA). The same mice were euthanized, and organs/tissues (tumor, heart, liver, kidney, spleen, and lung) were harvested for *ex vivo* bioluminescence imaging. Bioluminescence intensity (BLI) in the tumor *in vivo* and organs/tissues *ex vivo* was quantified by IVIS Lumina imaging software (Perkin-Elmer, USA).

### Immunological analysis

Tumor tissues were obtained from CT-26 tumor-bearing mice on Day 13 after treatment with IR plus CRT-targeting L-ASNases. Tissues were weighted and then cut into small pieces (~1 mm^3^). The samples (0.1 g) were incubated with 1 mL digestion solution [collagenase type IV (1.0 mg/mL) and deoxyribonuclease I (50 μg/mL) in RPMI 1640] at 37 °C for 30 min in a water bath shaker. Samples were passed through 100 μm and 40 μm nylon mesh filters to obtain single cell suspensions, and then mixed at a ratio of 1:10 with RBC lysis buffer (eBioscience, USA) at 25 °C for 5 min to remove red blood cells. The obtained cells were stained with different fluorophore-conjugated antibodies specific for CD4^+^ T cells (CD3^+^CD4^+^), CD8^+^ T cells (CD3^+^CD8^+^), and Tregs (CD4^+^CD25^+^Foxp3^+^) at 4 °C for 1 h. DCs were isolated from crushed tumor tissues using a MojoSort™ mouse dendritic cell isolation kit (BioLegend, USA), and stained at 4 °C for 1 h with fluorochrome-conjugated antibodies specific for MHCII and CD86. Fluorescent signals were measured in a flow cytometer (BD Biosciences, USA) and analyzed using FlowJo software (TreeStar, USA). The concentrations of TNF-α and IL-1β in the supernatants of tumor lysates were measured using ELISA kits specific for each cytokine.

### Immunohistochemical analysis of tumor tissues

Tumor tissues were harvested from mice and fixed overnight at 4 °C in 4% PFA. After washing three times with PBS, the tissues were transferred to a 30% sucrose solution and incubated overnight at 4 °C for cryoprotection. The tissues were then mounted in optimal cutting temperature (OCT) embedding compound (Thermo Scientific, USA) and frozen at -80 °C. Tissue slices (6 μm thick) were cut using a microtome (Thermo Scientific, USA) and mounted on glass slides. The slices were stained with biotinylated anti-Ki67, anti-CD31, and anti-ASNS antibodies, and visualized using an HRP/DAB (ABC) detection IHC kit. Immunohistochemistry images were obtained under a CKX41 optical microscope (Olympus, Japan). Signal intensities were quantified using ImageJ software (NIH, USA).

### Statistical analysis

All data are expressed as the mean ± standard error of the mean (SEM). Statistical analysis was performed using Prism 5.0 software (GraphPad, USA). Survival analysis was performed using the Kaplan-Meier method and the log-rank test. *P*-values < 0.05 were considered significant (*), those < 0.01 very significant (**), and those < 0.001 extremely significant (***).

## Results

### Binding efficiency of CRT-targeting monobodies

**Figure 1:**
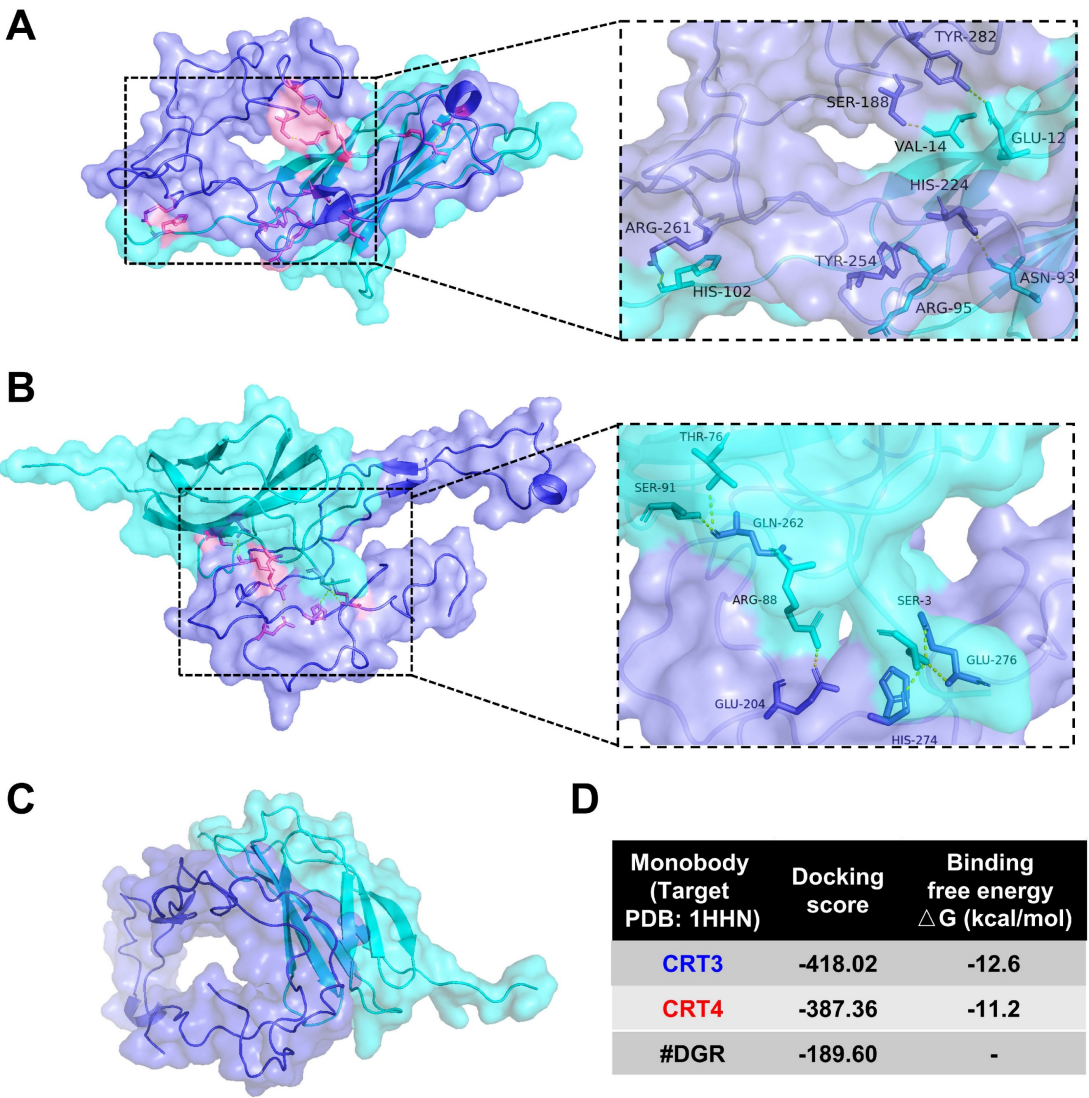
** Molecular docking analysis of binding between CRT and its specific monobodies.** CRT and monobody were presented as blue-violet and cyan colors, respectively. Their binding sites were presented as pink (see also the expanded dashed square). Amino acid residues assumed to be involved in binding were indicated. **(A)** Docking pattern model of CRT3. **(B)** Docking pattern model of CRT4. **(C)** Docking pattern model of #DGR. **(D)** Docking score and binding free energy of each monobody.

Previously, we assessed the specific binding of CRT-targeting monobodies 48, 49. Here, we analyzed monobody binding to CRT using an *in silico* molecular docking model and LigPlot^+^ 2.2.4 software (**Figure 1**). The simulated docking model showed that residues His-102, Arg-95, Asn-93, Val-14, and Glu-12 in the CRT3 monobody formed hydrogen bonds with the CRT residues Arg-261, Tyr-254, His-224, Ser-188, and Tyr-282, respectively (**Figure 1A**). Similarly, Ser-91, Thr-76, Arg-88, and Ser-3 in the CRT4 monobody formed hydrogen bonds with Glu-262, Glu-204, Glu-276, and His-274, respectively, in CRT (**Figure 1B**). By contrast, none of the residues of control #DGR mediated specific binding to CRT (**Figure 1C**). The binding energies against CRT were -12.6 kcal/mol for CRT3 and -11.2 kcal/mol for CRT4 (**Figure 1D**). Consistent with our previous reports 48, 49, these data indicate that the monobodies bound specifically to CRT with high affinity.

### IR-induced cell death increases ecto-CRT and PD-L1 expression

**Figure 2:**
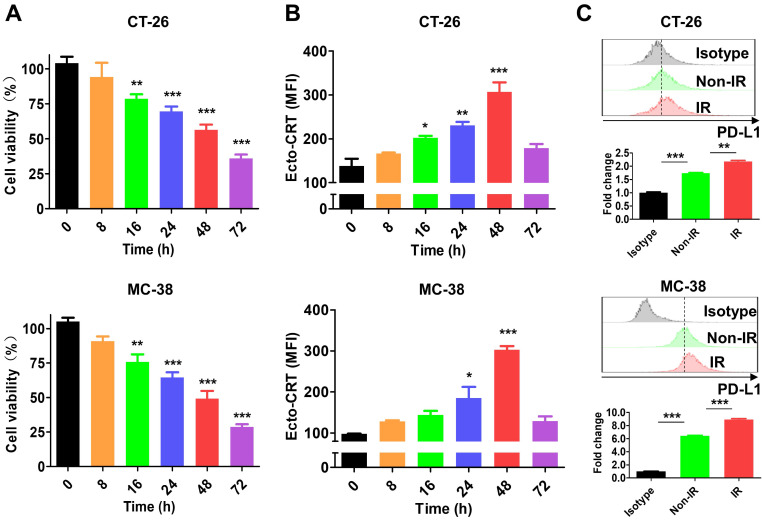
** Characterization of IR effect in tumor cells.** After tumor cells (CT-26 and MC-38) were treated with 10 Gy IR, markers (ecto-CRT and PD-L1) were stained with specific antibodies and assessed by flow cytometry at the indicated times. **(A)** Viability of CT-26 and MC-38 cells. **(B)** Exposure of CRT on CT-26 and MC-38 cells. **(C)** Expression of PD-L1 on CT-26 and MC-38 cells. PD-L1 expression was assessed at 48 h after IR treatment. Upper panels, histograms; bottom panels, relative fluorescence intensities (fold change). Fluorescence intensity of the samples was calculated relative to that of isotype-stained cells. Non-IR, untreated; IR, IR-treated. Data were presented as the mean ± SEM (n = 3). * p < 0.05, ** p < 0.01, *** p < 0.001, and ns, non-significant. MFI, mean fluorescence intensity.

A previous study showed that 4-10 Gy IR triggers tumor cell death within 48 h 51. In this study, treatment of CT-26 and MC-38 cells with 10 Gy IR for 0-72 h decreased cell viability in a time-dependent manner (**Figure 2A**) and upregulated the ICD marker ecto-CRT, which showed the highest expression level at 48 h in both IR-treated cell lines (**Figure 2B**). At the time, ecto-CRT levels in CT-26 and MC-38 cells were increased 2.22-fold and 3.09-fold, respectively, and was higher than that in Non-IR control cells. These data are consistent with previous results 51, 52, and indicate that the cytotoxic effect of IR is mediated by ICD. We also assessed PD-L1 expression by IR-treated tumor cells at 48 h. The levels were 1.25-fold higher in CT-26 and 1.39-fold higher in MC-38 than those in Non-IR controls (**Figure 2C**). These data were consistent with previous reports that expression of various immune checkpoints increased on the surface of IR-treated cells 53, 54.

### CRT-targeting L-ASNases promotes IR-mediated ROS generation

**Figure 3:**
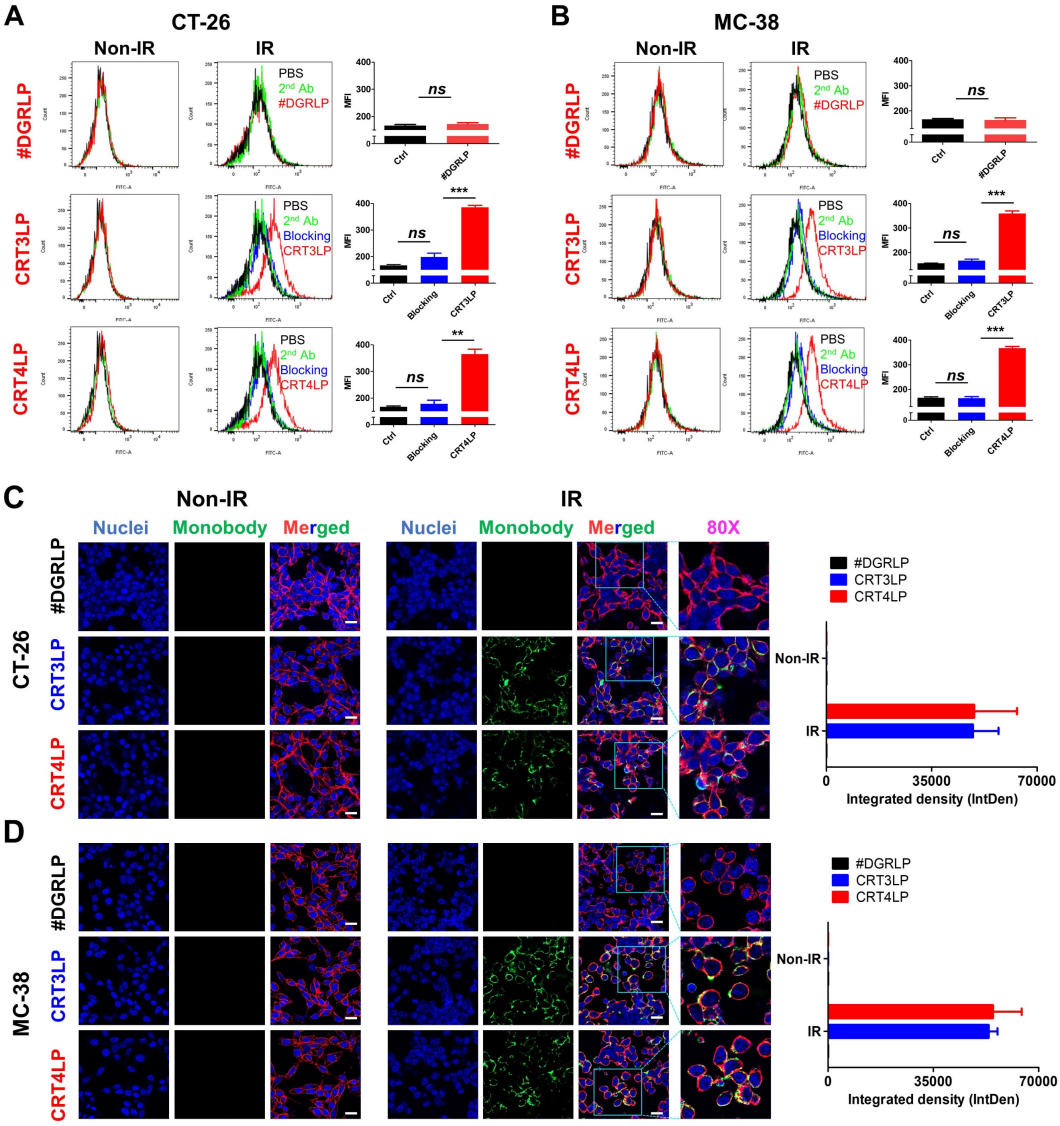
** Binding of CRT-targeting L-ASNases to IR-treated tumor cells.** For flow cytometry, tumor cells were treated without (Non-IR) or with (IR) 10 Gy IR and cultured for 48 h. Cells were then incubated with CRT-targeting L-ASNases (CRT3LP and CRT4LP), followed by anti-His and FITC-conjugated secondary antibodies. To block ecto-CRT, IR-treated cells were pre-treated with an anti-CRT antibody prior to CRT-targeting L-ASNases staining. #DGRLP was used as a negative control. PBS, unstained; 2^nd^, FITC-conjugated secondary antibody only (Ctrl). MFI, mean fluorescence intensity. For immunohistochemistry, cells were cultured on dishes and stained with CRT-targeting L-ASNases monobodies (green), DAPI (nucleus, blue), and WGA (cell membrane, red). **(A)** Flow cytometry profiles and quantitative assessment of CT-26 cells stained with CRT-targeting L-ASNases. **(B)** Flow cytometry profiles and quantitative assessment of MC-38 cells stained with CRT-targeting L-ASNases. **(C)** Immunofluorescence images and semiquantitative analysis of CT-26 cells stained with CRT-targeting L-ASNases. **(D)** Immunofluorescence images and semiquantitative analysis of MC-38 cells stained with CRT-targeting L-ASNases. Scale bar, 50 µm. Merged, triple-color merged images; 80×, 80-fold enlarged images. Data were presented as the mean ± SEM (n = 3). * p < 0.05, ** p < 0.01, *** p < 0.001, and ns, non-significant.

In a previous study, we reported that CRT monobody-L-ASNase-PAS200 conjugates (CRT3LP and CRT4LP) specifically bind to ecto-CRT, depleted Asn and more increased cell death in tumor cells treated with chemotherapy 49. In this study, we assessed whether CRT3LP and CRT4LP bound specifically to IR-treated CT-26 (**Figure 3A**) and MC-38 (**Figure 3B**) cells. After 10 Gy IR treatment and 48 h culture, CRT3LP and CRT4LP, but not control L-ASNase #DGRLP, efficiently bound only to IR-treated cells. Pre-treatment with an anti-CRT antibody (blocking group) significantly inhibited the binding of CRT3LP and CRT4LP to IR-treated cells. These data were consistent with the immunofluorescence analyses (**Figure 3C-D**).

**Figure 4:**
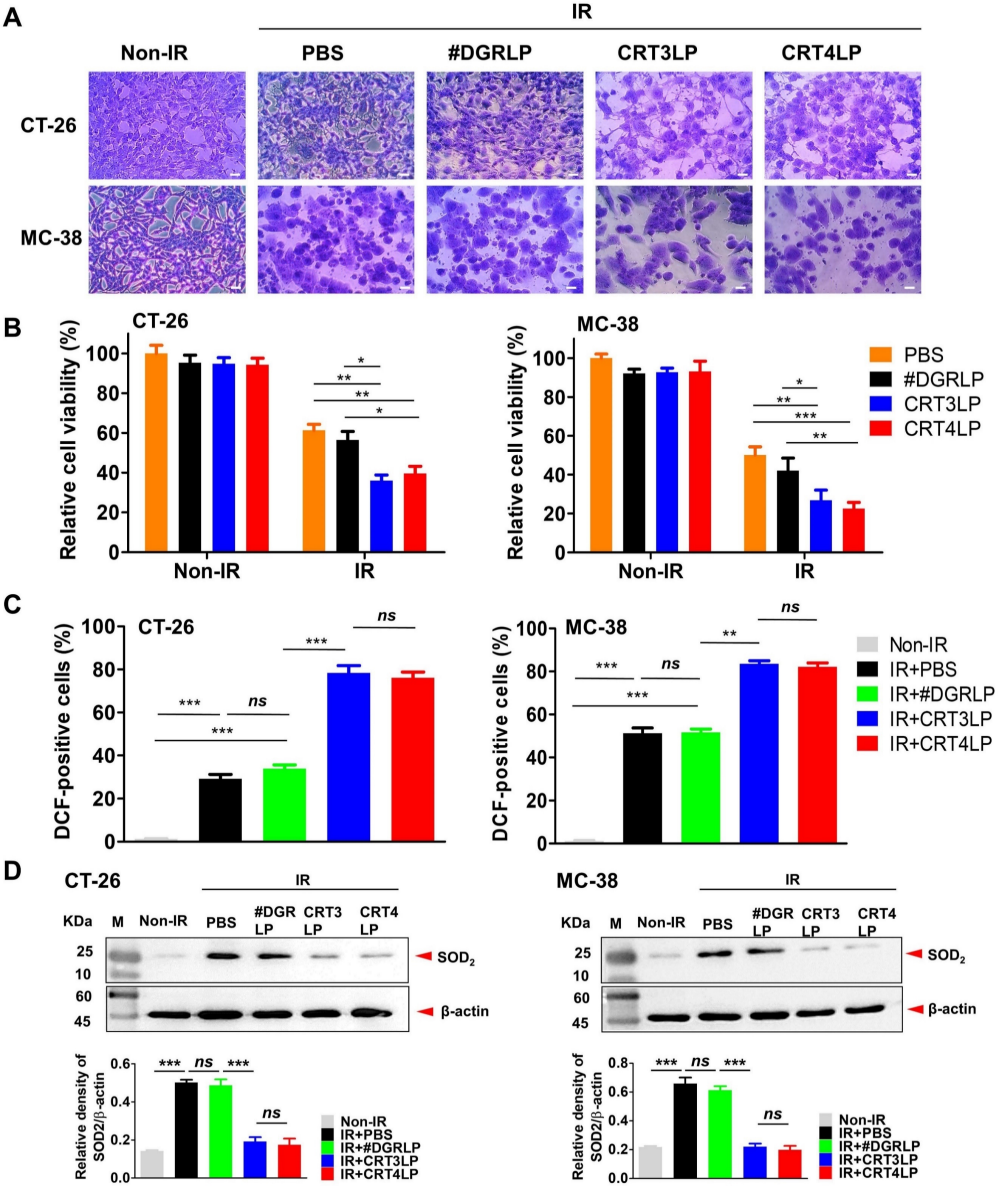
** CRT-targeting L-ASNases enhances cytotoxicity and ROS generation in IR-treated tumor cells.** Tumor cells (CT-26 and MC-38) were treated with 10 Gy IR, cultured for 24 h, and washed. CRT-targeting L-ASNases (1 IU/mL) were then added to the culture for 24 h. Cell viability, ROS, and SOD_2_ were measured. Non-IR, IR-untreated; IR, IR-treated. **(A)** Representative microscopy images of tumor cells co-treated with IR and CRT-targeting L-ASNases. Cells were stained with crystal violet. Scale bar = 20 μm. **(B)** Viability of CT-26 and MC-38 cells. **(C)** Levels of intracellular ROS in CT-26 and MC-38 cells. Cells were stained with DCF and ROS levels were measured by flow cytometry. The intensity of the sample was calculated relative to that of the Non-IR control (%). **(D)** Expression of SOD_2_ in CT-26 and MC-38 cells. SOD was assessed by western blotting (upper panel), and band intensity was quantified and normalized to that of β-actin (bottom panel). Data were presented as the mean ± SEM (n ≥ 3). * p < 0.05, ** p < 0.01, *** p < 0.001, and ns, non-significant.

Next, we assessed whether IR-induced cytotoxicity was enhanced by CRT-targeting L-ASNases in CT-26 and MC-38 tumor cells. Cells treated with 10 Gy IR were cultured for 24 h, washed, and exposed to 1 IU/mL CRT-targeting L-ASNases for 24 h. IR-treated cells (IR + PBS) and those treated with IR and #DGRLP (IR + #DGRLP) showed various morphological changes, including cell enlargement, increased nuclear-to-cytoplasmic ratio, and prominent nucleoli (**Figure 4A**). These changes were more prominent in cells treated with IR and CRT-targeting L-ASNases (IR + CRT3LP and IR + CRT4LP). These observations were consistent with the results of cell viability assays (**Figure 4B**). CRT3LP and CRT4LP had little effect on the viability of Non-IR cells, whereas they had a marked effect on IR-treated cells. IR-treated CT-26 cells showed a viability of 61.3% with PBS, 56.4% with #DGRLP, 36.0% with CRT3LP, and 39.6% with CRT4LP. The effects of CRT3LP and CRT4LP were similar in IR-treated MC-38 cells. These data indicate that CRT3LP and CRT4LP caused an approximately 1.5-fold greater decrease in the viability of IR-treated cells than PBS or #DGRLP.

There were no significant changes in ASNS levels in CT-26 and MC-38 cells treated with L-ASNase (**Figure S1**). Treatment with both IR and CRT3LP did not significantly change ASNS levels in these cell lines, as determined by western blotting (**Figure S2**). Immunohistochemistry results showed that ASNS expression was not significantly upregulated in CT-26 tumor tissues treated with IR and CRT3LP (**Figure S3**). These data indicate that CRT-targeting L-ASNases insufficient to affect ASNS expression even in the presence of IR.

L-ASNase promotes the generation of excess ROS to induce metabolic stress and mitochondrial injury, resulting in the inhibition of mTORC1 signals related to apoptotic cell death and cell cycle arrest 22, 55. We hypothesized that cellular ROS generation would increase in the IR + CRT3LP and IR + CRT4LP groups. The results showed that ROS levels of IR + CRT3LP (78.3%) and IR + CRT4LP (76.1%) groups were significantly higher than those in IR + #DGRLP (33.9%) group in CT-26 cells (**Figure 4C**, left). The increase in ROS levels were associated with decreased expression of SOD_2_, a mitochondrial dismutase that decreases ROS levels (**Figure 4D**, left). SOD_2_ levels increased in IR treatment (IR + PBS and IR + #DGRLP) but decreased in CRT-targeting L-ASNases treatment (IR + CRT3LP and IR + CRT4LP) until ones in Non-IR cells. Similar results were observed in MC-38 cells (**Figure 4C**, right and **4D**, right). These data indicate that CRT3LP and CRT4LP downregulated SOD_2_ expression in IR-treated tumor cells, leading to an increase in cellular ROS levels.

### Targeting of CRT-specific monobodies to IR-treated tumors

**Figure 5:**
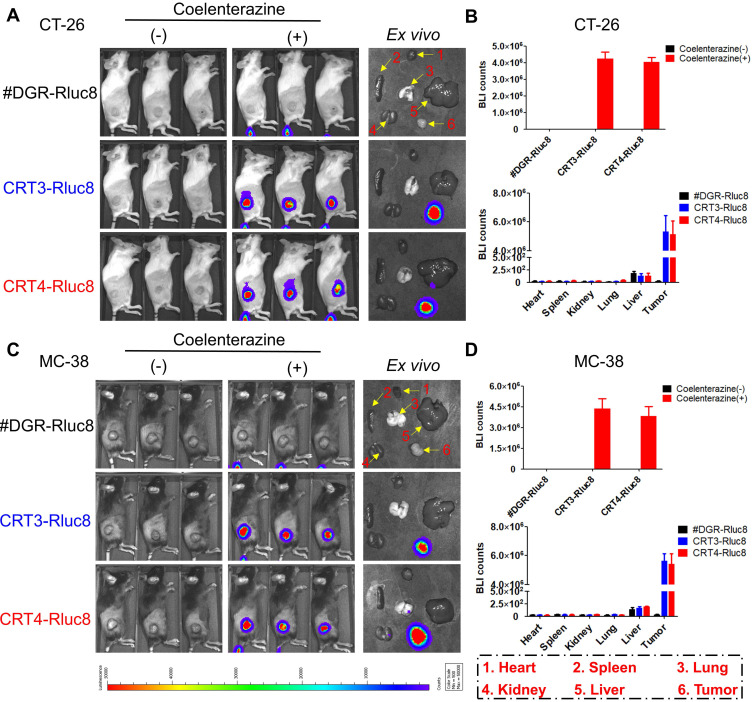
***In vivo* targeting of CRT-targeting monobodies in IR-treated tumor-bearing mice.** BALB/c and C57BL/6 mice (n= 3/group) were implanted *s.c.* with CT-26 and MC-38 tumor cells. When tumor size reached approximately 100 mm^3^, the mice were IR-treated (6 Gy). After 5 days, 60 µg of Rluc8-conjugated CRT-specific monobodies (CRT3-Rluc8 and CRT4-Rluc8, #DGR-Rluc8 as control) were injected *i.v.* and bioluminescence images were obtained after administration (or not) of coelenterazine. (-), without coelenterazine; (+), with coelenterazine. **(A)** *In vivo* and *ex vivo* bioluminescence images of CT-26 tumor-bearing mice. A representative *ex vivo* image was shown. **(B)** Quantification of bioluminescence signals in CT-26 tumor-bearing mice. Upper panel, *in vivo* bioluminescence; bottom panel, bioluminescence in each *ex vivo* organ. **(C)*** In vivo* and *ex vivo* bioluminescence images of MC-38 tumor-bearing mice. A representative *ex vivo* image was shown. **(D)** Upper panel, *in vivo* bioluminescence; bottom panel, bioluminescence of each *ex vivo* organ. Data were presented as the mean ± SEM

Previous studies showed that 6 Gy IR was a dose to suppress but did not completely eradicate tumors in mice 56, 57. To assess the targeting potential for IR-treated tumors, CRT3-Rluc8 and CRT4-Rluc8 were *i.v.* injected into CT-26 and MC-38 tumor-bearing mice treated with 6 Gy IR (**Figure 5**). After administration of Rluc8 substrate coelenterazine in CT-26 tumor-bearing mice, bioluminescence signals were specifically observed around tumors at Day 5.5 (**Figure 5A**). By contrast, no signals were detected in mice injected with control #DGR-Rluc8. These results were consistent with those obtained from *ex vivo* organs/tissues (**Figure 5B**). The bioluminescence signals were strongly detected in tumors and not or less in other tissues in the mice treated with CRT3-Rluc8 and CRT4-Rluc8 but not #DGR-Rluc8. Similar results were observed in MC-38 tumor-bearing mice (**Figure 5C-D**). These results indicate that CRT3 and CRT4 monobodies bind specifically to ecto-CRT on tumors treated with RT, indicating their potential as imaging agents to assess the efficacy of radiotherapy.

### CRT-targeting L-ASNases enhance RT-mediated antitumor immune responses in CT-26 tumor-bearing mice

**Figure 6:**
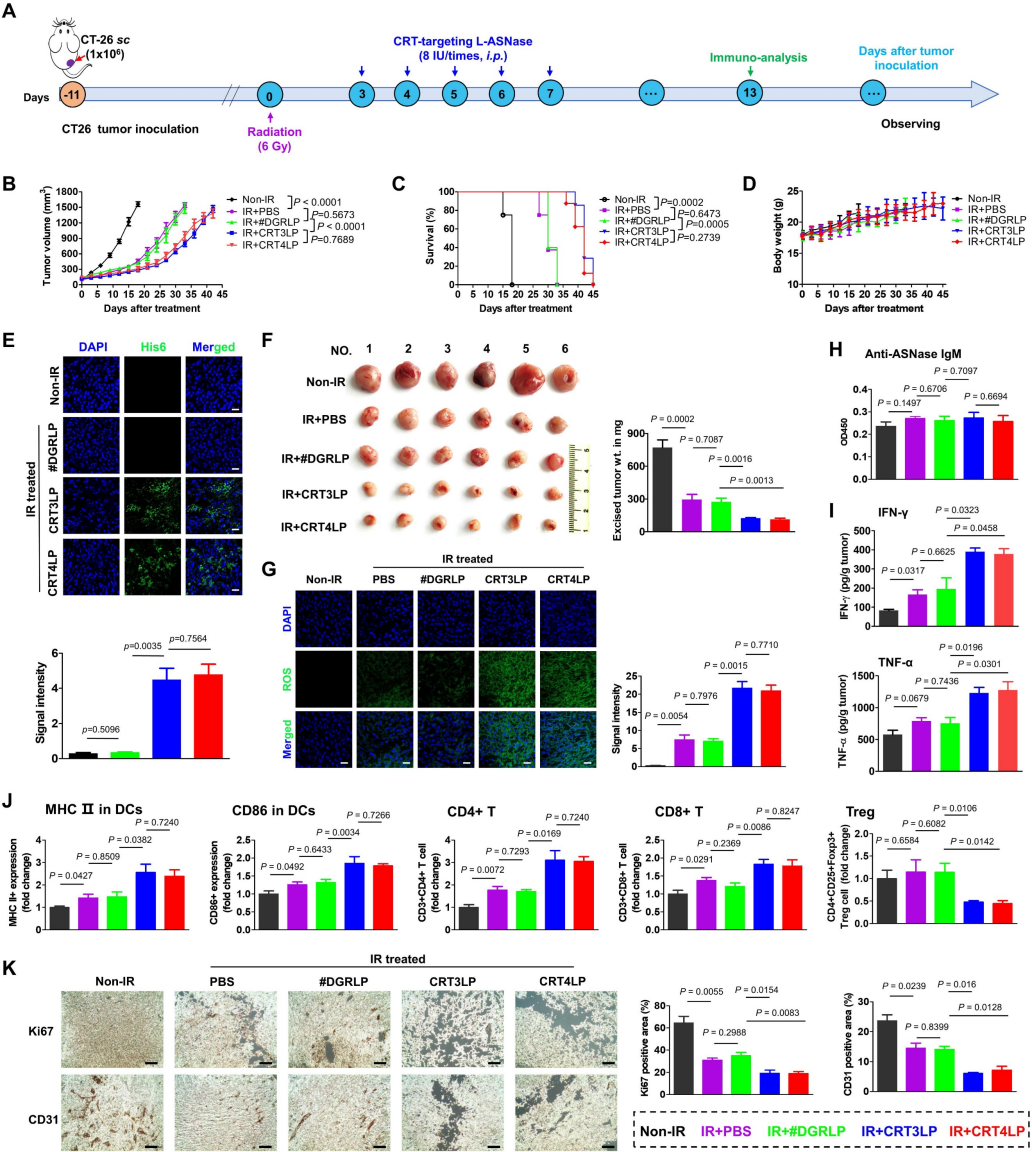
** Antitumor immune responses induced by CRT-targeting L-ASNases in CT-26 tumor-bearing mice treated with IR. (A)** Experimental scheme showing the CT-26 tumor model. CT-26 tumor cells (1 × 10^6^) were implanted *s.c.* into BALB/c mice at Day -11. The mice were then treated without or with 6 Gy IR (Non-IR or IR) at Day 0. CRT-targeting L-ASNases (8 IU) were *i.p.* injected every day from Day 3 to Day 7. Immunological analysis of tumor tissues was performed at Day 13. Tumor growth was observed until Day 45. **(B)** Average tumor growth curve for each group (n ≥ 5). **(C)** Kaplan-Meier survival curves (n ≥ 5). **(D)** Changes in body weight (n ≥ 5). **(E)** Immunohistochemistry images of tumor tissues at Day 5 (n = 3). Upper panel, representative images; bottom panel, quantification of fluorescence signals. Tumor tissues were stained with an anti-His tag antibody (green) and DAPI (nucleus, blue). Images were taken at 40 × magnification. **(F)** Average weight of *ex vivo* tumors at Day 13 (n = 6). Left panel, representative *ex vivo* tumor images; right panel, average tumor weight.** (G)** ROS levels in tumor tissues (n = 4). Left panel, representative confocal images of tumor tissues treated with an anti-His antibody (green); right panel, quantification of fluorescence signals. **(H)** Levels of anti-ASNase IgM in mouse serum at Day 13 (n = 4). **(I)** Levels of pro-inflammatory cytokines (IFN-γ and TNF-α) in tumor tissues at Day 13 (n = 4). **(J)** Population of tumor-infiltrating immune cells at Day 13 (n = 4). DCs, CD4^+^ T, CD8^+^ T, and Treg cells were obtained from tumor tissues and analyzed by flow cytometry. **(K)** Changes in tumor proliferation markers at Day 13. Tumor tissue sections were stained with antibodies specific for Ki67 and CD31, which are markers of proliferation and angiogenesis, respectively. After obtaining immunohistochemistry images (left panel), each positive signal was counted (right panel). Scale bar = 100 μm. Data were presented as the mean ± SEM (n ≥ 4).

The antitumor activity of CRT-targeting L-ASNases was evaluated in CT-26 tumor-bearing mice treated with 6 Gy IR (**Figure 6**). The results showed that tumor growth suppression was comparable between IR + PBS and IR + #DGRLP mice treated for 45 days (**Figure 6B** and **Figure S4**). Also, tumor suppressions were similarly enhanced in IR + CRT3LP and IR + CRT4LP mice more than IR + PBS and IR + #DGRLP mice. Moreover, the survival rates were extended for over 10 days in IR + CRT3LP and IR + CRT4LP mice more than those in IR + PBS and IR + #DGRLP mice (**Figure 6C**). Significant variations were not observed in body weights of all treated mice in the mean times (**Figure 6D**). These data indicate that CRT-targeting L-ASNases increased the antitumor efficacy of IR. Tumor targeting by CRT3LP and CRT4LP was assessed by incubation with anti-His tag antibody and imaging by confocal microscopy at Day 5 (**Figure 6E**). The fluorescence signals were clearly observed in the tumor tissues of IR + CRT3LP and IR + CRT4LP mice, but not in those of Non-IR and IR + #DGRLP mice. These data demonstrate that CRT3LP and CRT4LP specifically target ecto-CRT on IR-treated tumors.

Tumor weights were measured at Day 13 (**Figure 6F**). The average tumor weights were 769.5 mg in Non-IR, 295.3 mg in IR + PBS, 273.0 mg in IR + #DGRLP, 124.2 mg in IR + CRT3LP and 113.7 mg in IR + CRT4LP mice, respectively. Based on the results of Figure 4 showing that CRT-targeting L-ASNases bound to IR-treated tumor cells and induced metabolic stress, IR-treated tumor tissues were stained to detect ROS at Day 13 (**Figure 6G**). ROS signals were almost negligible in Non-IR tumor tissues, whereas high staining intensity was observed in all IR-treated tumor tissues. Notably, the signals were markedly higher in IR + CRT3LP and IR + CRT4LP tumors than those in IR + PBS and IR + #DGRLP tumors. The relative signal intensities were over 2.5-fold higher in IR + CRT3LP and IR + CRT4LP tumors than in IR + PBS and IR + #DGRLP tumors. This indicated that CRT-targeting L-ASNases synergistically enhanced ROS production in IR-treated tumors. No significant changes in serum IgM levels were observed in the tested mice at Day 13, indicating that CRT-targeting L-ASNases did not elicit primary antibody responses in IR-treated tumor-bearing mice (**Figure 6H**).

Next, we analyzed antitumor immune responses in these mice. First, we measured the levels of pro-inflammatory cytokines in tumor tissues at Day 13 (**Figure 6I**). IFN-γ levels in IR + PBS and IR + #DGRLP mice increased significantly more than Non-IR control mice. The levels were even higher in IR + CRT3LP and IR + CRT4LP mice (1.99-fold and 1.93-fold *vs.* IR + #DGRLP). TNF-α levels were similarly changed (1.63-fold in IR + CRT3LP and 1.69-fold in IR + CRT4LP* vs.* IR + #DGRLP). Then, we performed flow cytometry analysis to assess immune cell populations in tumors at Day 13. Mature DCs (MHC II^+^ and CD86^+^) in all IR-treated mice compared with Non-IR control mice (**Figure 6J**), with higher levels in IR + CRT3LP and IR + CRT4LP mice than in IR + PBS and IR + #DGRLP mice. This indicated that CRT-targeting L-ASNases promoted DC maturation in IR-treated tumors. Because DC maturation is related to antitumor responses by T cells 58, we next assessed changes in T cell populations (**Figure 6J**). CD4^+^ T cells increased in all IR-treated tumor tissues, but were higher in IR + CRT3LP and IR + CRT4LP mice than in IR + #DGRLP mice. Similar changes were observed in cytotoxic CD8^+^ T cells. Additionally, immunosuppressive Treg cells most markedly decreased in IR + CRT3LP and IR + CRT4LP mice. Furthermore, we assessed expression of CD31 and Ki67 markers in the tumors (**Figure 6K**), which reflect their angiogenesis and proliferation, respectively 59, 60. Levels of CD31 and Ki67 were high in Non-IR tumors, but lower in all IR-treated tumors. These reductions were greater in IR + CRT3LP and IR + CRT4LP tumors than those in IR + PBS and IR + #DGRLP tumors.

Taken together, these results indicate that CRT-targeting L-ASNases synergistically enhanced the antitumor immune responses induced by RT.

### Antitumor effects of combination treatment with CRT-targeting L-ASNases and αPD-L1 in RT-treated tumor-bearing mice

**Figure 7:**
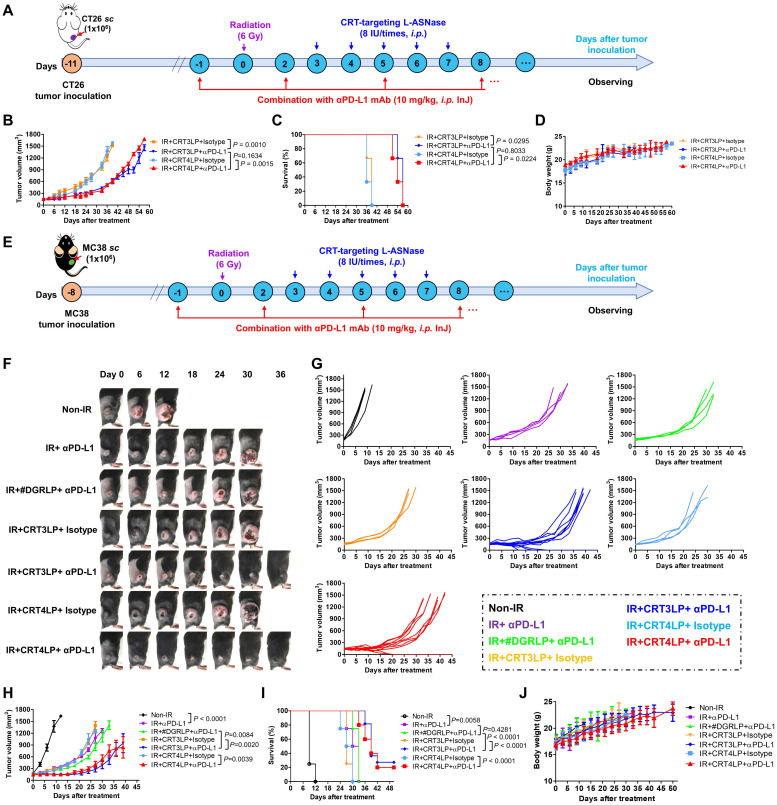
** Antitumor efficacy of combination with CRT-targeting L-ASNases and αPD-L1 treatment in IR-treated tumor-bearing mice. (A)** Experimental scheme for the CT-26 tumor models. CT-26 tumor cells (1 × 10^6^) were implanted* s.c.* into Balb/C mice at Day -11. The mice were then treated with IR (6 Gy, once), CRT-targeting L-ASNases (8 IU per mouse, five times), and αPD-L1 (10 mg/kg, every 3 days). An isotype antibody was used as a control for αPD-L1. **(B)** Changes in CT-26 tumor size. **(C)** Kaplan-Meier survival curves. **(D)** Changes in body weight of CT-26 tumor-bearing mice. **(E)** Experimental scheme for the MC-38 tumor model. MC-38 tumor cells (1 × 10^6^) were implanted *s.c.* into C57BL/6 mice at Day -8. The mice were then treated as described in **(A)**. **(F)** Representative images of MC-38 tumor-bearing mice taken at the indicated days. **(G)** Changes in tumor size. **(H)** Individual tumor growth curves in each group. **(I)** Kaplan-Meier survival curves. **(J)** Changes in body weight. Data were presented as the mean ± SEM (n ≥ 3).

Immune checkpoint molecules such as PD-L1 allow tumor cells to escape immune surveillance during RT, and their function is blocked by ICIs, resulting in enhanced RT efficacy 43, 61. Because IR upregulated PD-L1 expression (**Figure 2**), we hypothesized that the combination of CRT-targeting L-ASNases with an αPD-L1 antibody would further enhance the therapeutic efficacy of RT. To assess this, we *i.p.* administered αPD-L1 to CT-26 tumor-bearing mice treated with 6 Gy IR and CRT-targeting L-ASNases (**Figure 7A**). Tumor suppression was more significant in IR + CRT3LP + αPD-L1 and IR + CRT4LP + αPD-L1 mice than in IR + CRT3LP + isotype and IR + CRT4LP + isotype mice (**Figure 7B** and **Figure S5**). Moreover, survival rates were extended for over 15 days in IR + CRT3LP + αPD-L1 and IR + CRT4LP + αPD-L1 mice more than ones in IR + CRT3LP + isotype and IR + CRT4LP + isotype mice (**Figure 7C**). There were no significant changes in the body weight of tested mice during the treatment period (**Figure 7D**).

Finally, the antitumor efficacy of CRT-targeting L-ASNases was investigated in MC-38 tumor-bearing mice treated with IR. Similar to the effects in CT-26 tumor-bearing mice, CRT-targeting L-ASNases significantly enhanced the therapeutic efficacy of IR in these mice. Tumor growth was suppressed and survival rates were increased to a greater extent in IR + CRT3LP and IR + CRT4LP mice than in Non-IR, IR + PBS and IR + #DGRLP mice (**Figure S6A-D**). No significant changes in body were observed in the tested mice (**Figure S6E**). The antitumor efficacy of combination treatment with CRT-targeting L-ASNases and αPD-L1 was also assessed in MC-38 tumor-bearing mice treated with IR (**Figure 7E**). Tumor suppression was greater in mice exposed to combination treatment (IR + CRT3LP + αPD-L1 and IR + CRT4LP + αPD-L1) than in control mice (IR + CRT3LP + isotype and IR + CRT4LP + isotype). It was noted that tumors were eradicated completely in some combination-treated mice (3 of 11 in IR + CRT3LP + αPD-L1 and 2 of 10 in IR + CRT4LP + αPD-L1) (**Figure 7F-I**). Treatment with αPD-L1 suppressed tumor growth more significantly than CRT-targeting L-ASNases (IR + αPD-L1 *vs.* IR + CRT3LP + isotype and IR + CRT4LP + isotype) (**Figure 7H**) without significant body weight changes in all tested mice (**Figure 7J**). These data indicate that αPD-L1 acted synergistically with CRT-targeting L-ASNases to enhance the antitumor efficacy of RT.

## Discussion

L-ASNase is used to treat blood cancers such as leukemia, although it is not approved for the treatment of solid tumors 19, 62. We previously showed that CRT-targeting L-ASNases improve the antitumor efficacy of ICD-inducing chemotherapy against solid tumors 49.

Here, we showed that CRT-targeting L-ASNases enhanced antitumor efficacy of RT, and this effect is mediated by enzymatic activity and immune modulation in the tumor microenvironment. CRT-targeting L-ASNases specifically bound to ecto-CRT on tumor cells treated with IR and depleted Asn to enhance ROS production, which was associated with decreased expression of SOD_2_. L-ASNases also increased antitumor immune responses such as pro-inflammatory cytokine production, DC maturation and T cell activation and decreased Treg cells to prevent the immunosuppressive response in IR-treated tumors. IR upregulated the expression of PD-L1 on tumors to promote evasion from immune surveillance, which was blocked with αPD-L1. These findings shed light on the antitumor mechanisms mediated by IR, CRT-targeting L-ASNases and αPD-L1 (**Scheme 1**).

Nevertheless, antitumor immune responses are not sufficient to completely eradicate tumors despite effective combination treatments. In this study, tumor-bearing mice were treated with 6 Gy IR to assess the antitumor effects of combination with CRT-targeting L-ASNases and αPD-L1. The use of higher doses of IR may lead to tumor cell death and higher ecto-CRT levels. Under these conditions, higher amounts of CRT-targeting L-ASNases could be targeted to IR-treated tumors. It is also possible that CRT-targeting L-ASNases do not induce sufficient antitumor immune response in RT-treated tumors. To overcome these challenges, new recombinant proteins are being developed to carry CRT-specific monobodies, L-ASNases, and immune modulators such as pro-inflammatory cytokines and adjuvants. We previously reported the expression of an IL15-FlaB conjugate secreted from *S. typhimurium* for the treatment of cancer 47.

The use of ASNS level as a predictive indicator for L-ASNase cancer therapy is controversial 32. In many tumor cells, including those used in this study (CT-26 and MC-38), IR induces ICD, resulting in the exposure of CRT on the cell surface. However, some tumor cells are resistant to IR, and CRT exposure with multiple dependent factors 63, 64. For example, breast cancer 4T1 and melanoma B16F10 cells weak undergo ICD in response to IR (data not shown). Therefore, CRT-targeting L-ASNases are only effective in combination with RT in IR-sensitive cancers, and combination treatment should be indicated only after verifying that IR induces ICD.

In summary, CRT-targeting L-ASNases (CRT3LP and CRT4LP) are effective against solid tumors treated with RT, and their use in combination with PD-L1 blockade synergistically increases their efficacy.

## Conclusion

CRT-targeting L-ASNases and αPD-L1 are promising therapeutic agents when used in combination with IR.

## Supplementary Material

Supplementary figures.

## Figures and Tables

**Table 1:** Antibodies used in this study

Antibody (Ab)	Company/catalog number	Remarks
β-Actin (13E5) Rabbit mAb	Cell Signaling/4970L	1:1000 dilution
PE anti-mouse CD274 (B7-H1, PD-L1) Ab	BioLegend/155404	5 μg/mL
Recombinant anti-6X His tag Ab	Abcam/ab245114	1:500-2000 dilution
Goat anti-rabbit secondary Ab, Alexa Flour 488	Thermo Scientific/A-11008	5 μg/mL
Calreticulin (D3E6) XP® rabbit monoclonal Ab, PE	Cell Signalling/19780S	1:50 dilution
Anti-calreticulin monoclonal Ab	Thermo Fisher/MA5-15382	1:500 dilution
Wheat germ agglutinin (WGA)-AF555 conjugate	Thermo Scientific/W32464	1:5000 dilution
Recombinant anti-SOD2/MnSOD Ab	Abcam/ab68155	1:1000 dilution
Anti-rabbit secondary Ab, HRP	Invitrogen/31460	1:2000 dilution
Goat anti-mouse IgM (heavy chain) secondary Ab, horseradish peroxidase (HRP)	Thermo Scientific/62-6820	1:2000 dilution
Recombinant anti-Ki67 Ab [SP6]	Abcam/ab16667	1 µg/ml
Recombinant anti-CD31 Ab	Abcam/ab182981	1:100 dilution
CD86 (B7-2) monoclonal Ab (GL1), FITC	eBioscience™/11-0862-81	0.125 µg/test
MHC Class II monoclonal Ab (HIS19), APC	eBioscience™/17-0920-82	0.25 µg/test
Anti-CD3 monoclonal Ab, APC	eBioscience™/17-0038-42	0.25 µg/test
Anti-CD8a monoclonal Ab, PE	eBioscience™/12-0081-82	0.25 µg/test
Anti-CD4 monoclonal Ab, FITC	BioLegend/100412	0.25 µg/test
Anti-CD4 monoclonal Ab, APC/Cy7	Invitrogen/A15384	0.25 µg/test
Anti-CD25 monoclonal Ab, PE	BioLegend/102008	0.25 µg/test
Anti-Foxp3 monoclonal Ab, Alexa Fluor® 488	BioLegend/126405	0.25 µg/test
InVivoMAb anti-mouse PD-L1 (B7-H1)	BioXcell/BE0101	10 mg/kg
InVivoMAb rat IgG2b isotype control	BioXcell/BE0090	10 mg/kg
Asparagine Synthetase Antibody	Cell Signalling/20843S	1:1000 dilution
