# Supplementary material for: The combination of calreticulin-targeting L-ASNase and anti-PD-L1 antibody modulates the tumor immune microenvironment to synergistically enhance the antitumor efficacy of radiotherapy
Source: Theranostics. 2024 Jan 20;14(3):1195–211. doi: 10.7150/thno.90376 (PMC10845202; doi:10.7150/thno.90376)
Supplement: Supplementary file 1 — Supplementary figures. [file thnov14p1195s1.pdf]

Supporting information

The combination of calreticulin-targeting L-ASNase and anti-PD-L1 antibody modulates the tumor immune microenvironment to synergistically enhance the antitumor efficacy of radiotherapy

Ying Zhang[1,9], Venu Akhil[1], Ho Seong Seo[2], Hae Ran Park[2], Soo Hyun Kim[3,4], Sung-Hwan You[1,5], Zhipeng Liu[6], So-young Kim[1,5], Rukhsora D. Sultonova[1,7,8], Jung-Joon Min[1,5*], and Yeongjin Hong[1,3,5*]

[1]*Institute for Molecular Imaging and Theranostics, Department of Nuclear Medicine, Chonnam National University Medical School and Hwasun Hospital, Jeollanam-do, Republic of Korea*

[2]*Research Division for Radiation Science, Korea Atomic Energy Research Institute, Jeollabuk-do, Republic of Korea*

[3]*Department of Microbiology, Chonnam National University Medical School, Jeollanam-do, Republic of Korea*

[4]*Department of Laboratory Medicine, Chonnam National University Medical School and Chonnam National University Hospital, Gwangju, Republic of Korea*

[5]*CNCure Biotech, Inc, Jeollanam-do, Republic of Korea*

[6]*Brain Tumor Research Laboratory, Biomedical Research Institute, Chonnam National University Hwasun Hospital, Jeollanam-do, Republic of Korea*

[7]*New Uzbekistan University, Tashkent, Uzbekistan*

[8] *Republican Oncology Research Center Tashkent Region Branch, Tashkent, Uzbekistan*

[9]***Current affiliation:*** *State Key Laboratory of Drug Research, Molecular Imaging Center, Shanghai Institute of Materia Medica, Chinese Academy of Sciences, Shanghai, 201203, China*

*Correspondence to Drs. Yeongjin Hong and Jung-Joon Min

Yeongjin Hong, Ph.D.

Department of Microbiology, Chonnam National University Medical School

264, Seoyang-ro, Hwasun-eup, Hwasun-gun, Jeollanam-do, 58128, Republic of Korea

E-mail: yjhong@jnu.ac.kr

Jung-Joon Min, M.D., Ph.D.

Department of Nuclear Medicine, Chonnam National University Medical School and Hwasun Hospital

264, Seoyang-ro, Hwasun-eup, Hwasun-gun, Jeollanam-do, 58128, Republic of Korea

E-mail: jjmin@jnu.ac.kr

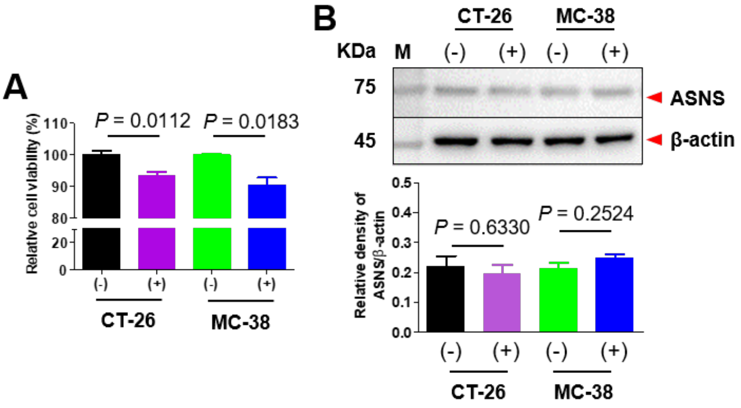

Figure S1. ASNS expression in CT-26 and MC-38 tumor cells treated with L-ASNase. CT-26 and MC-38 cells (4×10^4) cultured overnight were treated without (-) or with (+) L-ASNase (1 IU/mL) for 24 h. (**A**) Viability of tumor cells. Relative viability percentages were compared between (+) groups and (-) groups. (**B**) ASNS expression levels. Western blot analysis of protein expression using anti-ASNS or anti-β-actin antibodies (upper panels). Band intensities were measured and expressed relative to β-actin. Data are presented as the mean ± SEM (n = 3).

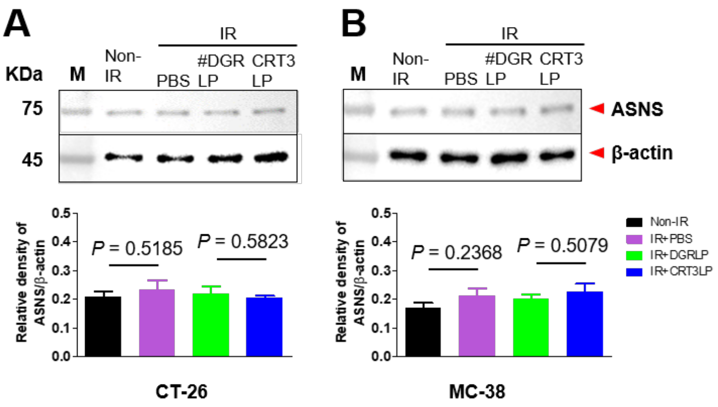

Figure S2. ASNS expression in tumor cells treated with IR and CRT3LP. CT-26 and MC-38 cells were treated with 10 Gy IR and cultured for 24 h. CRT3LP or #DGRLP (1 IU/mL) were added for 4 h. Cells were washed and cultured for an additional 24 h. (**A**) ASNS expression in CT-26 cells and (**B**) in MC-38 cells. ASNS expression was assessed by western blotting using anti-ASNS antibody and anti-β-actin antibodies as controls (upper panels), and band intensities were quantified. The amounts of ASNS were normalized to that of β-actin (bottom panels). Data are presented as the mean ± SEM (n = 3).

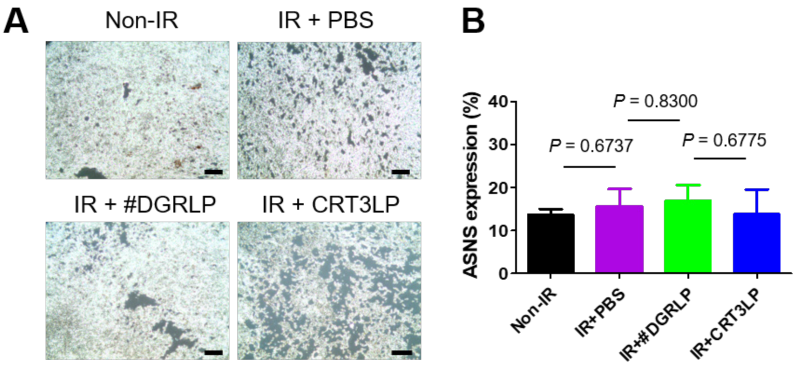

Figure S3. Immunohistochemistry of ASNS in CT-26 tumor tissues treated with IR and CRT3LP. CT-26 tumor tissues, which were the same as those shown in Figure 6K, were stained with anti-ASNS antibody. (**A**) Immunohistochemistry images. Scale bar = 100 µm. (**B**) Quantification analysis. Positive dots were counted. Data are presented as the mean ± SEM (n = 3).

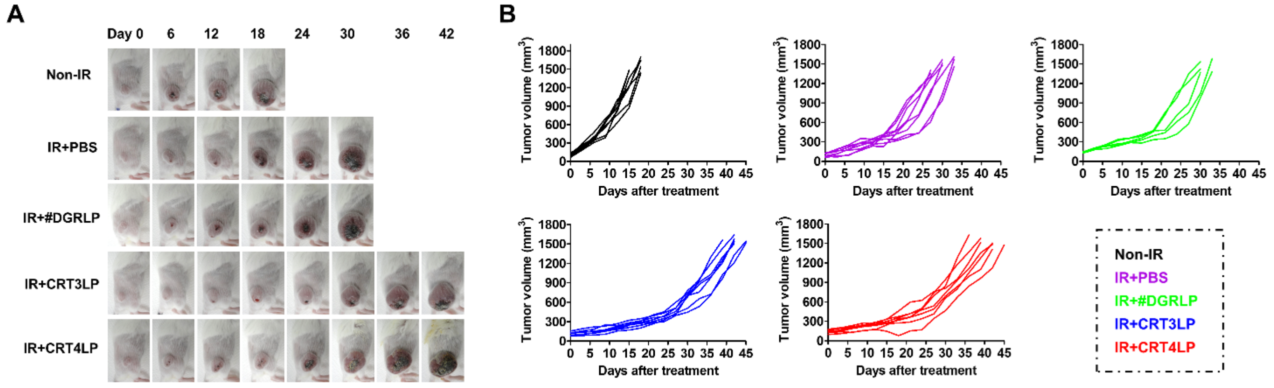

Figure S4. Antitumor effect of CRT-targeting L-ASNases in CT-26 tumor-bearing mice treated with IR. (**A**) Representative images of CT-26 tumor-bearing mice. (**B**) Individual tumor growth curves for each group. Data are presented as the mean ± SEM ($n \geq 5$).

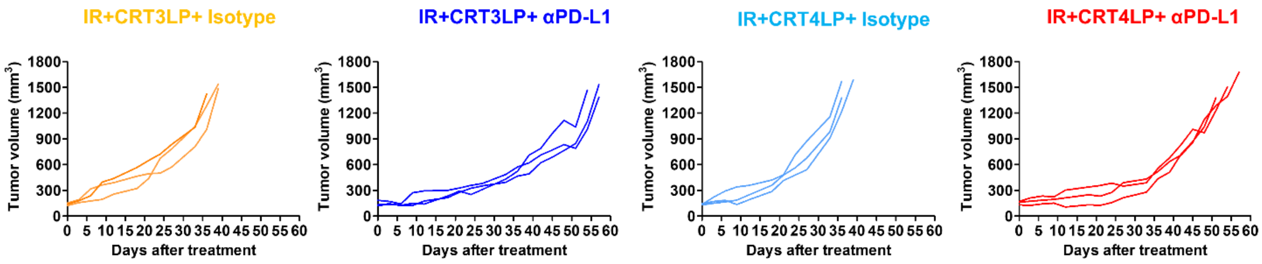

Figure S5. Individual tumor growth curves after combination therapy with CRT-targeting L-ASNase and αPD-L1 in CT-26 tumor-bearing mice treated with IR (n = 3).

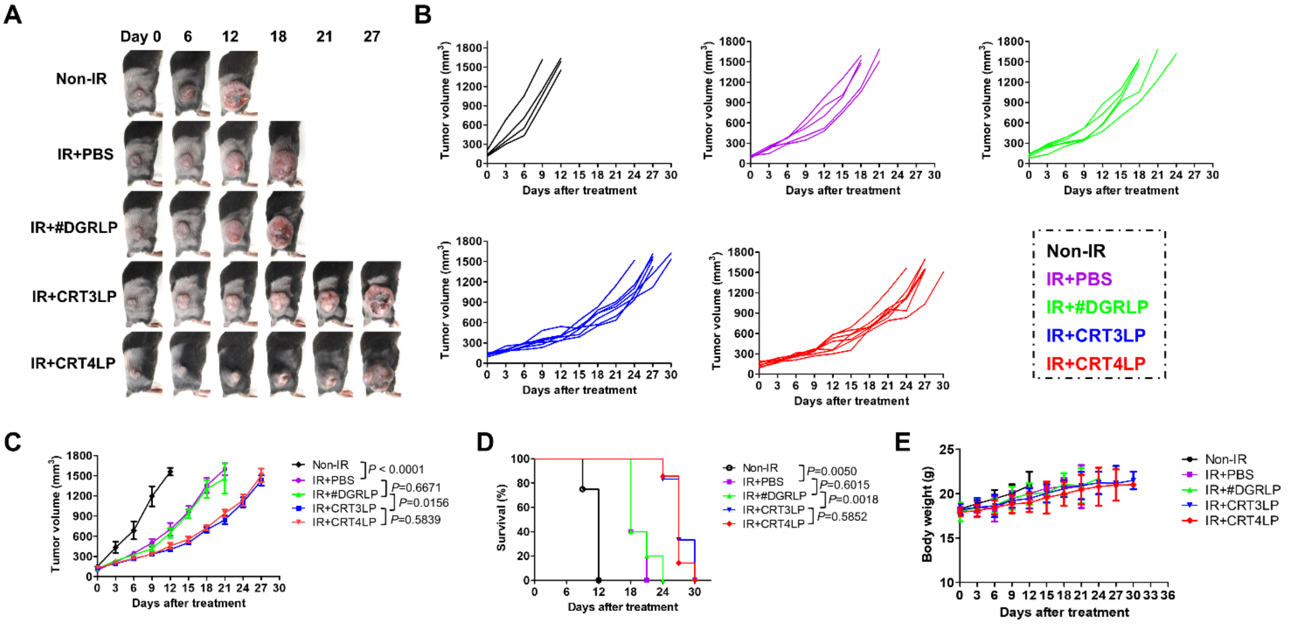

Figure S6. CRT-targeting L-ASNases synergistically enhance antitumor efficacy in MC-38 tumor-bearing mice treated with IR. (**A**) Representative images of MC-38 tumor-bearing mice. (**B**) Individual tumor growth curves for each group. (**C**) Changes in tumor size. (**D**) Kaplan–Meier survival curves. (**E**) Changes in body weight. Data are presented as the mean ± SEM (n ≥ 4).
